# Epigenome and three-dimensional genome architecture remodeling during NDM29-mediated retro-transformation of neuroblastoma cells

**DOI:** 10.1371/journal.pone.0327466

**Published:** 2025-07-31

**Authors:** Francesca Baldini, Aldo Pagano, Lama Zeaiter, Paolo Bianchini, Hawraa Zbeeb, Alberto Diaspro, Laura Vergani

**Affiliations:** 1 Nanoscopy, Istituto Italiano Tecnologia, Genova, Italy; 2 DIMES, Department of Experimental Medicine, University of Genoa, Genova, Italy; 3 IRCCS Ospedale Policlinico San Martino, Genova, Italy; 4 DISTAV, Department for the Earth, Environment and Life Sciences, University of Genova, Genova, Italy; 5 DIFILAB, Department of Physics, University of Genova, Genova, Italy; Università di Pisa: Universita degli Studi di Pisa, ITALY

## Abstract

Neoplastic transformation of mammalian cells involves intricate interactions between genetic, epigenetic and architecture modifications of the nucleus. Neuroblastoma is a malignant pediatric tumor with high biological and clinical heterogeneity representing a challenging model of study. We aimed to explore the changes in genome architecture and epigenetics being associated with neuroblastoma malignancy. We employed the neuroblastoma cell line SKNBE2 overexpressing the ncRNA NDM29 to differentiate from highly malignant into neuron-like cells. By 3D confocal microscopy, we explored the nuclear architecture (volume, elongation, compactness, and chromatin density). Using super-resolution microscopy (STED) and histone H3 immunolabelling we assessed the epigenetic rearrangement, and by enzyme-linked immunoassay the global DNA methylation. Then we assessed the mRNA expression of the main epigenetic modifying enzymes by quantitative PCR, and the expression of NF-κB-regulated genes by cDNA microarray. Compared to malignant NB cells, the NDM29-overexpressing cells, assuming a neuron-like phenotype, exhibited smaller and more elongated nuclei, redistribution of H3K9-acetylated and -methylated chromatin domains and DNA hypermethylation. In line with these results, in neuron-like cells the acetyltransferase KAT2A and the DNA methyltransferase DNMT1 were up-regulated, while most of NF-κB-regulated genes were down-regulated. Our findings reveal modifications of the nuclear structure and epigenome during neuroblastoma retro-transformation induced by NDM29 overexpression, with impacts on gene expression. These results offer potential insights into better understanding the mechanism of neuroblastoma malignancy in terms of chromatin rearrangements, opening exciting prospects for prognostic and therapeutic approaches with a focus on the nuclear level.

## Introduction

Three-dimensional (3D) genome architecture plays a central role in maintaining physiological cellular functions and determining cell fate [1,2]. Genome structure exhibits a hierarchical organization of multiscale structural units ranging from chromosome territories to active and inactive chromatin compartments. Indeed, genomic rearrangements are a common feature of cancer cells leading to inactivation/activation of specific genes [3].

Chromatin can be classified into euchromatin and heterochromatin based on its transcriptional activity and condensation: euchromatin contains transcriptionally active genes and is loosely arranged, while heterochromatin is tightly condensed and primarily comprises transcriptionally-silent genes [4].

Both chromatin condensation and genome architecture are finely regulated by epigenetic mechanisms. Epigenetics defines the processes that alters heritable gene expression beyond the information coded by the DNA sequence [5]. Epigenetics consists of the addition/removal of specific chemical groups to the histone tails and to the DNA itself. While histones are subjected to a variety of post-translational modifications, at the present state the only epigenetic modification of DNA well documented is the methylation of the CpG islands [6]. DNA methylation is catalyzed by the DNA methyltransferase enzymes (DNMTs) that counts five members, with the DNMT1 being the most abundant isoform in human cells [7]. The histone methylation at lysine 9 on histone 3 is catalyzed by the histone-lysine N-methyltransferase 1 and 2 (EHMT1 and EHMT2) leading to silencing of gene expression [8]. The lysine acetyltransferase 2A (KAT2A) catalyzes the histone acetylation at lysine 9 on histone 3 [9].

Alterations in epigenome are recognized to drive the malignant transformation of cells and a bidirectional epigenetic crosstalk between cancer cells and the tumor microenvironment are decisive for cancer onset and progression [10]. It is accepted that cancer is predominantly initiated by genetic modifications while predisposition and progression are epigenetically dominated [11]. Therefore, modifications of the epigenome and nuclear architecture could act in onset and progression of neuroblastoma (NB), an embryonal tumor of the sympathetic nervous system characterized by clinical and molecular heterogeneity. NB originates from cells of the neural crest failing to undergo a complete adrenergic differentiation and initiating a neoplastic process towards an aggressive and metastatic tumor [12]. Although aberrant epigenetic landscapes, such as abnormal DNA methylation patterns [13,14], or oncogenic super-enhancers [15], unravelling the pathogenic pathway of NB malignant progression associated with the 3D genome remains a challenge.

The non-coding RNAs (ncRNAs) have gained attention for their role in many cancers, including NB [16]. Our group developed a human functionally active neural network derived from human neuroblastoma cancer cells genetically engineered to overexpress the non-coding RNA NDM29 (Neuroblastoma Differentiation Marker 29), whose increased synthesis causes the differentiation toward a neuronal phenotype [17,18].

This study aims to unveil and characterize the possible alterations of the nuclear morphometry, genome architecture, chromatin condensation and epigenetics being correlated with the recovery of a neuron-like phenotype by highly malignant NB cells through the over-expression of NDM29 ncRNA. A better understanding of the molecular and cellular mechanisms underlying neuronal differentiation through nucleus remodeling might pave the way toward novel therapeutic procedures.

## Materials & methods

### Chemicals

All chemicals, unless otherwise indicated, were supplied by Sigma-Aldrich Corp. (Milan, Italy).

### Cell culture and treatments

To generate a neuron-like cell line from SKNBE2, a human neuroblastoma cell line, cells were genetically engineered to enhance the expression level of the ncRNA NDM29 by stable transfection using polyethyleneimine with pEGFP-N1 as control (hereafter referred to as Mock) or pEGFP-N1-NDM29 (hereafter referred to as S1.1) [19]. For antibiotic selection and the maintenance of transfected cells, 200 μg/mL geneticin (G418) (Roche, Milano, Italy) and 0.1 μL/mL puromycin (Sigma Aldrich, Milano, Italy) were used. S1.1 cell adopted neuron-like cell morphology and exhibit a late stage of differentiation toward a functional human neuronal lineage. Both Mock and S1.1 cells were grown in RPMI 1640 medium with 10% fetal bovine serum (FBS), 2mM L-glutamine and 100 U/L penicillin-streptomycin at 37°C, 5% CO2 atmosphere, 100% humidity.

### Cell fixation and immunostaining

For microscopical analyses, cells were grown on collagen-coated glass coverslips. At the end of treatments, cells were rinsed with phosphate-buffered saline (PBS) at pH 7.4, fixed with 4% paraformaldehyde in PBS for 15 min at 37°C, and washed 3 times with PBS. Then, cells were permeabilized using 1% BSA (albumin and bovine serum) in 0.5% Triton-PBS at 37°C for 15 min, and then further incubated in 3% BSA in 0.5% Triton-PBS at 4°C for 1 hour to block the non-specific binding of the antibodies during the immunostaining. To analyze homogeneous cell samples, we focused on G1 phase cells which were identified by immunolabeling for the CDT1 (chromatin licensing and DNA replication factor 1) a cyclin that accumulates during the G1 phase and is degraded at the beginning of the S phase thus representing a G1 phase marker [20]. To do so, after fixation and permeabilization, slides were incubated at 4°C overnight with the primary antibodies. For both confocal and STED imaging, we used the mouse anti-CDT1 1:200 (Catalog sc-365305, Santa Cruz) and Alexa Fluor 546 goat anti-mouse IgG 1:500 (Catalog A-11003, Thermofisher Scientific) as primary and secondary antibodies, respectively.

**For confocal microscopy** cells were stained with mouse anti-Tubulin β 3 (TUBB3) Antibody 1:500 (Catalog 801213, Biolegend) as a neuronal marker, using Alexa Fluor 546 goat anti-mouse IgG (1:500) as the secondary antibody, at room temperature for 1 hour.

**For STED microscopy** cells were stained for Lysin 9 on histone 3 trimethylated (H3K9Me3) or acetylated (H3K9Ac), alternatively, using the rabbit anti-H3K9Ac 1:200 (Catalog 49–1009, Invitrogen) or the rabbit anti-H3K9Me3 1:200 (Catalog ab8898, Abcam), respectively. After washing, the following secondary antibodies Alexa Fluor 546 goat anti-mouse IgG (1:500) and Aberrior Star 635P anti-rabbit 1:1000 (Catalog ST635P, Aberrior) were used at room temperature for 1 hour.

For both confocal and STED samples, the cells were incubated with Hoechst 33342 to detect the nuclear structure. Finally, slides were sealed using ProLong Diamond Antifade Mountant (Thermo Fisher Scientific).

### Confocal microscopy

The 3D confocal imaging of Hoechst-stained nuclei was employed to assess the nuclear morphology and chromatin organization. Images were acquired with a Nikon’s A1R MP confocal laser scanning microscope, using an Apo TIRF 60x 60/1.49 oil immersion objective lens (Nikon, Tokyo, Japan), sequentially and then merged. 512 x 512 pixel images were acquired with a pixel size of P(x,y) of 120 nm and a Pz of 150nm. Then, we employed an automatic tool, developed on ImageJ software [21], to obtain different morphological parameters out from the 3D fluorescence microscopy images collected. To separate the nucleus of interest both from the background and from the other nuclei that may be present in the field of view (ROI), a neural network has been trained with data manually labelled and then apply to the images. This automatic tool has been developed and recently published by our group [22]. We used this tool to extract different morphometric parameters, such as volume, compactness, elongation, and chromatin density [22]. In particular, the segmentation pipeline consists of two steps: separating nuclei from the background using intensity features, and splitting touching nuclei based on shape. A multi-threaded Random Forest classifier (200 trees, 8 threads) is used for voxel classification, trained either interactively or via a pre-trained model. Features include Gaussian blur, derivatives, Hessian, Laplacian, structure tensor, Canny edges, Difference of Gaussians, mean, and variance. The output is a binary mask with holes filled. To split overlapping nuclei, a watershed algorithm is applied with seeds from local maxima above a threshold. Nuclei closer than 10 voxels are not separated. The resulting mask defines the ROI. Morphological features and intensity-based metrics are computed. Volume is expressed in μm3, while compactness and elongation are expressed as dimensionless numbers. In detail, the compactness of a nucleus is calculated by dividing the volume by the surface area (V2/S3). Elongation measures the ellipticity of the object; it is calculated as the ratio between r1 and r2, which are the two main radii of the best fitted ellipsoid. In a sphere elongation value is equal to 1. Chromatin density was calculated as the ratio between the total intensity of the Hoechst signal and the nuclear volume.

### STED microscopy

Through stimulated emission depletion (STED) microscopy, we analysed histone’s modification markers associated with euchromatin and both constitutive and facultative heterochromatin [23–25]. We employed Leica Stellaris 8 Tau-STED microscope with HC PL APO CS2 100 × /1.40 oil immersion objective lens (Leica Microsystems, Mannheim, Germany). Emission depletion was accomplished with a 775 nm STED laser. The STED laser pulses are modulated at 80 mHz with a duration of 650 ± 100 psec, and spectral bandwidth (FWHM) < 1 nm. Excitation was provided by a white light laser tuned at the proper excitation wavelength for each sample. 512 x 512 pixel images were acquired with a pixel size of P(x,y) of 35 nm and a scan speed of 400 Hz. Micrographs were later processed using the software Leica Application Suite (LAS). The resolution of the STED images was calculated using the Fourier ring correlation (FRC) assay and is equal to 138 nm [26] (S1 Fig).

### Extraction of genomic DNA and MethylFlash global DNA

For each cellular sample, 5x10^6^ cells were collected by centrifugation, washed with PBS and stored at −80 °C until use. After thawing, the pellets were lysed in an optimized buffer and treated with Proteinase K to remove nucleases. Then, genomic DNA was extracted using the GeneAll ExgeneTM Cell SV mini (GeneAll) by using silica-binding technology, according to the manufacturer’s protocol [27]. UV-VIS spectrophotometry was used to test the quality and quantity of the extracted DNA using a Varian Cary-50Bio UV-VIS spectrophotometer (Agilent, Milan, Italy).

The global methylation level of the genomic DNA was measured using the MethylFlash™ Global DNA Methylation (5-methylcytosine, 5-mC) ELISA Easy Kit (Epigentek, USA), according to the manufacturer’s protocol. The amount of input DNA for each assay was 100 ng, to ensure optimal quantification. Five replicates were performed for each condition. Data are presented as the mean ± standard deviation of the replicates.

### RNA extraction and quantitative real-time polymerase chain reaction

Total RNA was isolated using a Trizol reagent. RNA concentration was quantified spectrophotometrically at 260 nm and purity was evaluated by measuring the ratio A260/A280. Total RNA was reverse transcribed using the RevertAid H Minus Reverse Transcriptase MULV (Thermo Fisher Scientific, Massachusetts, USA), according to the manufacturer’s suggestions, in a Master Cycler Personal (Eppendorf, Milan, Italy). Quantitative real-time PCR (qPCR) was performed in triplicate using iQ™ SYBR Green Supermix and CFX96™ Real-Time System (Biorad, Monza, Italy). All primer pairs were designed ad hoc starting from the coding sequences (http://www.ncbi.nlm.nih.gov/Genbank/GenbankSearch.html) and were synthesized by TibMolBiol custom oligosynthesis service (Genova, Italy). The relative quantity of target mRNA was calculated by using the comparative Cq method and was normalized for the expression of the GAPDH gene, used as a reference gene. The normalized expression of the target genes was thus expressed as a relative quantity of mRNA (fold induction) concerning Mock cells. Data were analyzed using a Bio-Rad CFX manager software. Primer pairs designed ad hoc starting from the coding sequences of Homo sapiens and synthesized by TibMolBiol, are listed in Table 1.

**Table 1:** The primer pairs of the studied genes.

Primer Name	Primer Sequence 5’- > 3’	Annealing Temperature (°C)	Accession ID
GAPDH Fwd	ACCCACTCCTCCACCTTTGACGC	60	NM_002046.3
GAPDH Rev	CGCTCCTGGGAAGATGGTGATGGG		
DNMT1 Fwd	GCTCTCCTGTTCCTCTTAATCC	60	NC_000019.10
DNMT1 Rev	GCAATCTCTGCCATCACG		
EHMT1 Fwd	GGAATTAGATGACAGTGACTTGGC	60	NC_000009.12
EHMT1 Rev	GGAGCACCTTGGCGAACAG		
KAT2A Fwd	GTCTGTTCACAAGGAAGAGG	56	NC_000077.7
KAT2A Rev	GCAAGAACATCTTTGAGAGC		
NDM29 Fwd	GGCAGGCGGGTTCGTT	60	
NDM29 Rev	CCACGCCTGGCTAAGTTTTG		

### Human NF-kB-regulated cDNA plate array

To profile the NF-κB-regulated genes in the Mock and S1.1 cells, we collected aliquots of 9 µg of total mRNA. Three sets of RNA were prepared, quantified, and then pooled by combining equal amounts (3 µg from each set).

Following the manufacturer’s instructions, we performed reverse-transcription of RNA into complementary DNA (cDNA) using labelled biotin-dUTP in the presence of reverse transcriptase enzyme. For NF-κB-regulated plate-based hybridization profiling, we utilized a 96-well cDNA plate array from Signosis Inc. (Sunnyvale, CA). The obtained cDNA samples were then allowed to hybridize into the array wells, enabling us to assess the targeted genes that were specifically captured on each plate well. These wells were pre-coated with gene-specific oligonucleotides and were detected using streptavidin-horseradish peroxidase conjugate.

The gene expression was quantified using a luminometer SpectraMax Mini Multi-Mode Microplate Reader (Molecular Devices, CA), which measured the luminescence of each gene in its respective well and expressed the results in relative light units. To ensure accuracy, background correction was performed using a blank well. Additionally, to account for variations in sample loading, the luminescent intensity of each gene was normalized using the cDNA of GAPDH as a reference.

### Statistical analysis

Data were expressed as means ± standard deviation (S.D.) of at least three independent experiments in triplicate. Statistical analysis was performed using Student’s t-test (GraphPad Software, Inc., San Diego, CA, USA).

## Results

### Nuclear morphometry remodeling in neuroblastoma cell transformation

**Fig 1:**
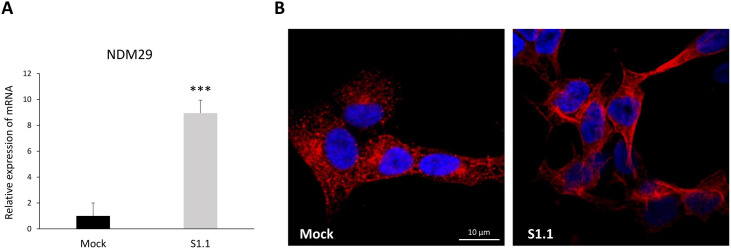
The cellular model of neuroblastoma retro-transformation. (A) The level of expression of the ncRNA NDM29 in a representative sample of Mock and S1.1 cells was evaluated by qPCR, using GAPDH as the internal control. Data are expressed as fold induction with respect to Mock. (B) A panel of representative images of cells stained simultaneously with mouse anti-tubulin β3 antibody (TUBB3) and Alexa Fluor 546, and with Hoechst 33342 (blue). Images were acquired with a Nikon’s A1R MP confocal laser scanning microscope, using an Apo TIRF 60x 60/1.49 oil immersion objective lens (Nikon, Tokyo, Japan), sequentially and then merged (Bar: 10 µm).

According to our model, the differentiation of highly malignant NB cells (SKNBE2 Mock) towards a neuron-like phenotype (SKNBE2-S1.1) is triggered by NDM29 ncRNA over-expression [14,17,19]. For accuracy assurance, before the experiments we tested all cells for NDM29 expression showing the expected marked up-regulation in the neuron-like phenotype (8.5-fold increase in S1.1 cells compared to Mock cells) (Fig 1A).

Tubulin β3 Class III (TUB β3) is a marker for neural differentiation, and we observed its constitutive presence in both Mock and S1.1 cells. However, the TUB β3 fluorescence was noticeably more organized and distributed in S1.1 cells, sign of a cytoskeletal remodeling being associated with the neuron-like phenotype (Fig 1B). Indeed, upon the NDM29 over-expression cells assume a neuronal phenotype (S1.1 cells) and the cell morphology results more elongated with the appearance of cellular extensions and intercellular connections typical of the neurons.

**Fig 2:**
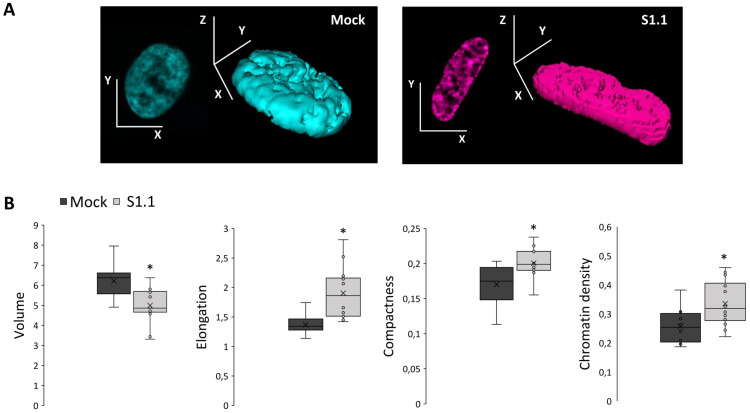
3D nuclear morphometry in neuroblastoma cell transformation. (A) The 3D nucleus reconstruction of a representative Mock (cyan) and S1.1 (magenta) cell by Confocal Microscopy. (B) The four boxplots represent the morphometric parameters being calculated on the 3D nuclei: volume (expressed in µm3), elongation (the ratio between the two main radii of the best fitted ellipsoid), compactness (the ratio between the volume and the surface area), chromatin density (the ratio between the sum of the nuclear fluorescence intensity and the volume). At least ten images for each condition have been quantified.

As the mechanical changes in the cytoskeleton are known to impact on cell nucleus [28], by 3D confocal microscopy of Hoechst-stained nuclei we investigated the nuclear remodeling occurring when the highly malignant Mock cells were retro-transformed (Fig 2A). As chromatin and nuclear organization change during cell cycle progression, we focused on cells in G1 phase that we identified by positive immunolabeling for the CDT1, a well-established G1-phase marker. The nuclear architecture was depicted by the following morphometric parameters: volume, elongation, compactness, and chromatin density (Fig 2B). The nuclei of neuron-like S1.1 cells, showed a smaller volume (486 µm3) and a more elongated shape (elongation of 1.86) compared to those of malignant Mock cells (volume of 638 µm3 and elongation of 1.34). These morphometric rearrangements reflected on the nuclear compactness which resulted higher in the neuron-like S1.1 cells (0.20) compared to the malignant Mock cells (0.18). Moreover, the changes in nuclear morphometry are associated with modifications in the chromatin density, which significantly increased in neuron-like S1.1 cells (0.319) with respect to malignant Mock cells (0.254) (Fig 2B). Therefore, the combined morphometric analyses of cell and nucleus unveil their coordinated remodeling when highly malignant NB cells recover a neuron-like phenotype.

### Epigenome rearrangement in neuroblastoma cell transformation

Taking advantage of super-resolution STED microscopy, we assessed the possible epigenome remodeling being associated with euchromatin and constitutive/facultative heterochromatin in cells at G1 phase. To this aim, we investigated the nuclear distribution of both the H39Me3 and the H3K9Ac chromatin domains, as they are preferentially associated with euchromatin and heterochromatin, respectively.

**Fig 3:**
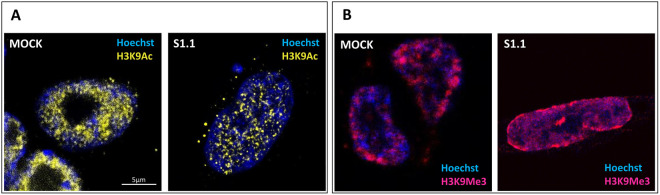
Epigenome rearrangement in neuroblastoma cell transformation. Representative STED images of Mock and S1.1 nuclei labelled for: (A) H3K9Ac by Aberrior Star 635P (yellow) and Hoechst (blue) and (B) H3K9Me3 by Aberrior Star 635P (magenta) and Hoechst (blue). Images were acquired sequentially and then merged, using a Leica Stellaris 8 Tau-STED microscope with HC PL APO CS2 100 × /1.40 oil immersion objective lens (Leica Microsystems, Mannheim, Germany). Emission depletion was accomplished with a 775 nm STED laser (Bar: 5 µm).

Regarding the histone H3 acetylation, the malignant Mock cells showed a rather diffuse H3K9Ac fluorescence, even if we could appreciate an empty central region as well as a preferential localization near the nuclear envelope, whereas in the neuron-like S1.1 cells, dotted/punctuate structures appeared (Fig 3A). Regarding the histone H3 methylation, in the malignant Mock cells the H3K9Me3 fluorescence was dispersed inside the nucleus, whereas in the neuron-like S1.1 cells the fluorescence was more intense and better organized in the proximity of the nuclear envelope (Fig 3B). Taken together our data show a redistribution of both the H3-methylated and H3-acetylated chromatin domains during the recovery of a neuron-like phenotype.

### DNA methylation pattern changes during neuroblastoma cell transformation

**Fig 4:**
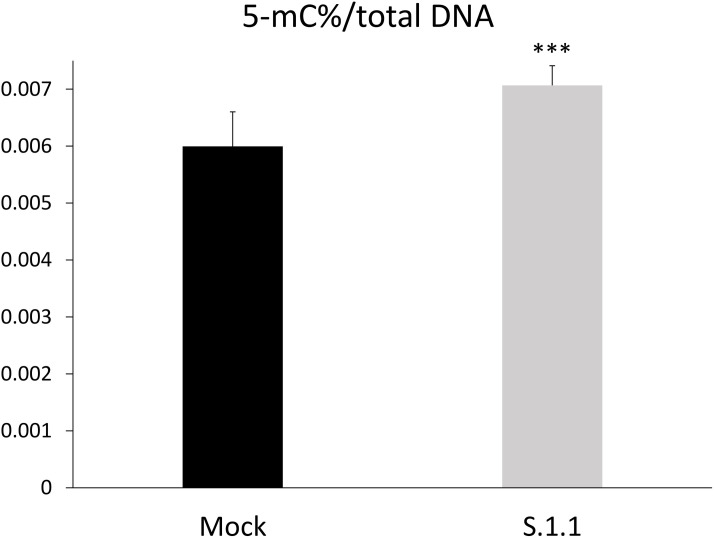
Global 5-methylcytosine methylation in neuroblastoma cell transformation. Quantification of 5-methylcytosines with respect to the total genomic DNA content in Mock and S1.1 cells using a MethylFlash™ Global DNA Methylation (5-methylcytosine, 5-mC) ELISA Easy Kit (Epigentek, USA). Statistical significance between groups was assessed by Student’s t-test. Symbols: Mock vs S1.1 ***p ≤ 0.001.

For a more complete investigation of the epigenome rearrangements associated to cell transformation, we assessed the global DNA methylation profile of the CpG sites taking advantage of ELISA assay. It is known that a global DNA hypomethylation occurs during neuroblastoma onset and progression [29]. Our results clearly indicate that, at the level of genomic DNA, the 5-mC content was significantly higher in the neuron-like S1.1 cells (+17%; p ≤ 0.001) compared to the Mock cells. (Fig 4). Therefore, the data indicate a general increase in DNA methylation being associated with the recovery of a neuron-like phenotype, as expected with the hypomethylated state of the more malignant stages.

### Expression profiles of the epigenetic modifying enzymes in neuroblastoma cell transformation

**Fig 5:**
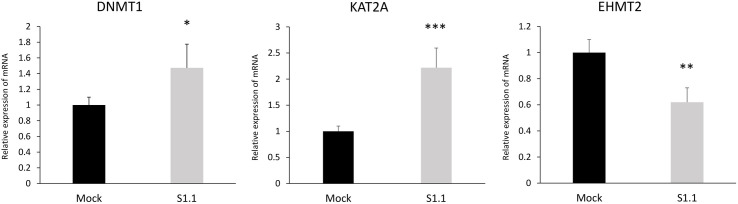
Expression of three epigenetic modifying enzymes genes in neuroblastoma cell transformation. The mRNA expression of DNMT1, KAT2A, and EHMT1 was evaluated by qPCR using GAPDH as the internal control. Data are expressed as fold induction with respect to controls. Statistical significance between groups was assessed by Student’s t-test. Symbols: Mock vs S1.1, *p ≤ 0.05, **p ≤ 0.01, ***p ≤ 0.001.

For a wider investigation about the epigenome remodelling, we measured the mRNA expression of the main epigenetic modifying enzymes by Real-Time RT-PCR. We focused on the enzymes DNMT1, EHMT2 and KAT2A. DNMT1 is a key DNA methyltransferase involved in cell differentiation and gene transcription [30]. Indeed, DNA methylation is a major epigenetic modification for gene silencing and altered DNA methylation is frequently observed in tumorigenesis [31,32]. EHMT2 is a histone methyltransferase for methylation of lysine 9 on histone H3, which is generally associated with transcriptional repression [33]. KAT2A is a histone acetyltransferase for the acetylation of lysine residues leading to increased chromatin accessibility and promoting gene transcription [34]. Of note, KAT2A acetylates also non-histone proteins and seems to act in both oncogenesis and genome stability regulation. Moreover, KAT2A acts as a repressor of the transcription factor NF-κB, which regulates a broad range of genes involved in inflammation, immune responses, cell proliferation, and apoptosis [35]. As shown in Fig 5, the mRNA expression of the DNA methyltransferase DNMT1 was significantly up-regulated in the neuron-like S1.1 cells with respect to the malignant Mock cells (1.47-fold induction; p ≤ 0.05). Also, the mRNA expression of the histone acetyltransferase KAT2A was up-regulated in the S1.1 cells with respect to the Mock cells (2.22-fold induction; p ≤ 0.001). By contrast, the mRNA expression of the histone methyltransferase EHMT1 was significantly down-regulated in the neuron-like S1.1 cells with respect to the Mock cells (0.62-fold induction; p ≤ 0.005).

### Expression profile of the main NF-kB-regulated genes in neuroblastoma cell transformation

**Fig 6:**
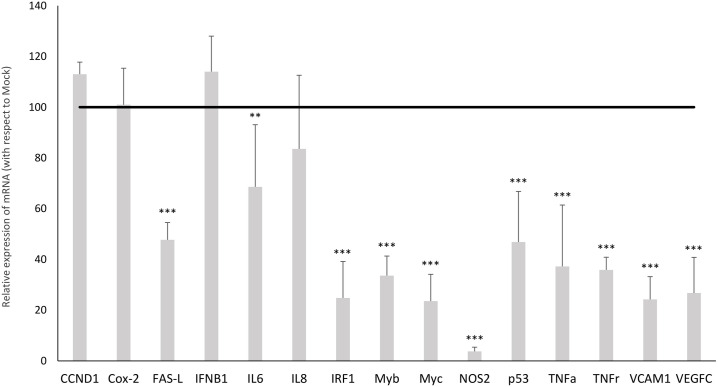
Expression profile of NF- κB-regulated genes in neuroblastoma cell transformation. The mRNA levels of genes (determined by Human NF-κB-Regulated cDNA Plate Array) in Mock and S1.1 cells. GAPDH was used as internal control. Data are expressed respect to Mock cells (the black line that represents 100%), while S1.1 are represented as grey histograms. Statistical significance between groups was assessed by Student’s T-test. Symbols: Mock vs S1.1, **p ≤ 0.01, ***p ≤ 0.001.

Although KAT2A is primarily a transcriptional activator of genes, it also functions as a repressor of NF-kappa-B (NF-κB) transcription factor [35]. Therefore, we assessed the expression profile of a pool of NF-κB-regulated genes by Elisa assay. In the neuron-like S1.1 cells, most of the tested genes were significantly down-regulated with respect to the Mock cells: the Fas ligand (FAS-L) of −52% (p ≤ 0.001), the interleukin 6 (IL6) of −31% (p ≤ 0.01), the interferon regulatory factor 1 (IIRF1) of −75% (p ≤ 0.001), the oncogenes MYB, MYC and p53 of −66%, −76% and −53%, respectively (p ≤ 0.001); the inducible nitric oxide synthase (NOS) of −96% (p ≤ 0.001), the cytokine TNFα of −63% (p ≤ 0.001), the TNFα receptor (TNFr) of −64% (p ≤ 0.001), the vascular cell adhesion molecule 1 (VCAM1) of −76% (p ≤ 0.001) and the vascular endothelial growth factor C, (VEGFc) of −73% (p ≤ 0.001) (Fig 6). Only few genes did not significantly change their expression between Mock and S1.1 cells: cyclin D1 (CCND1), cyclooxygenase-2 (Cox-2), interferon beta 1 (IFNB1) and interleukin 8 (IL8).

## Conclusions

Due to its remarkable biological and clinical heterogeneity, NB represents a challenge for understanding the mechanisms underlying the malignant transformation, at the cell level, beyond genetic mutations. The present study unveils chromatin and epigenome remodeling being associated with neuroblastoma malignancy by using an *in vitro* model where highly-malignant NB cells were genetically engineered to overexpress the ncRNA NDM29, which promotes their retro-transformation toward a neuron-like phenotype [17,26].

Our study revealed that when malignant NB cells recover a neuron-like phenotype, due to NDM29 over-expression, this event leads to a marked reorganization of the nucleus which becomes more elongated and smaller in volume with a global reduction in chromatin compactness. It is well known that nuclear shape results from deformations of the sub-nuclear components such as nuclear lamina and chromatin domains. Therefore, the next step has been to investigate if the nuclear remodeling observed during NB retro-trasnformation might be associated with epigenome modifications, primarily in DNA methylation and histone H3 acetylation and methylation, and eventually with changes in the transcriptional profile.

At first, we verified that the NDM29 overexpression in S1.1 cells is in line with previous data of our group [17,19]. Nuclear morphology is highly plastic and nuclear morphology changes are routinely observed in pre-neoplastic and malignant cancer tissues. Recent studies showed that cytoskeleton is an essential factor in reorganization of the nuclear shape and function although the mechanisms connecting the nuclear shape with cell morphology need to be clarified [36]. Therefore, by immunofluorescence analysis of tubulin β3 we observed the cytoskeletal reorganization sustaining the retro-transformation of malignant cells towards a neuron-like phenotype with elongated shape and cellular protrusions. Indeed, the neuron-like S1.1 cells exhibited an increased TUB β3 fluorescence with a redistribution in the cytoplasm, in line with the role of TUB β3 in neural differentiation [37]. Our results showing a reorganization of the cytoskeleton in the neuron-like phenotype strongly aligns with its alterations in tumor cells, where abnormal cytoskeleton contributes to the capacity of the tumor to resist chemotherapy and disseminate metastasis [38–40].

To investigate the possible nuclear rearrangement being associated with NB retro-transformation we reconstructed the 3D nucleus in both Mock and S1.1 cells by confocal microscopy. To exclude any influence of the cell-cycle phase on our analysis we focused on cells at G1 phase. The choice of focusing on this cell cycle phase takes the advantage of analyzing cells in their physiological activities such as RNA and protein production, and DNA repair before entering the next S phase. Moreover, G1 cells are rather homogeneous in terms of nuclear organization with intact nuclear envelope and functional chromatin organized in a stable and representative architecture.

In neuron-like cells S1.1 the nucleus becomes more elongated, and this ellipsoid-like re-shaping parallels with the more elongated and ellipsoidal shape observed for the whole cells. Of note, changes in nuclear size and shape are frequently observed in cancer cells [41], and many cancers are diagnosed and staged based on graded increases in nuclear size [42].

Changes in nuclear size and shape have often been linked to epigenetic- and nuclear envelope-associated factors [43]. Therefore, our study continued by investigating the histone H3 modifications typically being associated with euchromatin and heterochromatin domains [44].Since many chromatin modifications occur at length scales below the light diffraction limit, we exploited STED tunability to encode spatial details and assess the spatial rearrangement of chromatin domains rich in methylated or acetylated H3K9 residues.

It is well-known that heterochromatin segregates spatially from euchromatin and it is localized preferentially at the nuclear periphery, while euchromatin is more widely diffused and concentrates in the center of the nucleus [45]. When malignant Mock cells assume a neuron-like phenotype, we observed a clear redistribution of both H3K9-acetylated and -methylated chromatin in the nucleus, corresponding to euchromatin and heterochromatin, respectively. In the neuron-like S1.1 cells, the methylated domains (H3K9Me3) were more intense and preferentially localized closer to the nuclear envelope compared to malignant Mock cells where the fluorescence was more dispersed inside the nucleus. Also, acetylated domains (H3K9Ac) were redistributed in the S1.1 cells showing a reduced H3K9Ac fluorescence compared to Mock cells, together with the presence of dotted/punctuated structures in the central part of the nuclei compared. Our findings reveal a global redistribution of heterochromatin and euchromatin domains during the transition from a malignant to a neuron-like phenotype. The methylated (transcriptionally inactive) domains move towards the periphery of the nucleus, whereas the acetylated (transcriptionally active) domains accumulate in the center of the nucleus.

For the epigenome analysis we also assessed the DNA methylation of CpG sites using an ELISA assay. Our findings unequivocally demonstrate that the 5-mC content is substantially higher in S1.1 cells compared to the Mock cells. The significant global DNA hyper-methylation in neuron-like cells might sustain the restoration of a neuron-like phenotype. This finding was consistent with our result showing the up-regulation of the DNMT1 methyltransferases in the neuron-like S1.1 cells with respect to malignant Mock. Of note, the mammalian DNMT1 enzyme seems to regulate crucial physiological processes such as cell differentiation and gene transcription [7], and DNA methylation is the major form of epigenetic modification for the silencing of gene expression, often showing abnormalities during tumorigenesis [46].

For a deeper understanding, we quantified the expression of the main histone methyltransferases and acetyltransferases. Among the methyltransferase, EHMT catalyzes the demethylation of lysine 9 in histone H3 leading to the silencing of gene expression. Our data show a significant down-regulation of EHMT2 in S.1. cells with respect to malignant Mock. Indeed, EHMTs have been found to be up-regulated in certain cancers, and it has been linked to DNA repair processes and seems to be associated with chemoresistance in various cancer types [47]. Regarding the acetyltransferases, KAT2A promotes the acetylation of lysine residues, thereby facilitating the accessibility of transcriptional factors. In our model, we observed up-regulation of KAT2A mRNA expression in S1.1 cells compared to Mock cells, even though the H3K9Ac fluorescence signal resulted to be organized in confined dotted structures. This apparent discrepancy may be explained by taking into consideration that KAT2A catalyzes acetylation not only of the histone H3 tails, but of many other proteins, and the role of KAT2A in cancer is still under debate. Some recent publications have highlighted its potential oncogenic role as a chromatin modifier implicated in tumorigenesis and tumor progression [48], while other research suggested that KAT2A/2B may control genome stability [49].

Of note, in addition to its primary role as transcriptional activator KAT2A acts also as a repressor of the transcription factor NF-κB which regulates the expression of a panel of NF-κB-regulated genes [35]. Indeed, NF-κB controls many biological responses to physiological stress by regulating the expression of hundreds of genes reflecting on a wide range of cellular processes, including inflammation, immune responses, cell survival, cell proliferation, and apoptosis [50,51]. NF-κB dysregulation has been implicated in many diseases including some types of cancer. In some tumors, NF-κB stimulation seems to promote tumor cell proliferation and angiogenesis, suppress apoptosis and facilitate metastasis [52]. Compared to the malignant Mock cells, S1.1 cells exhibit a significant down-regulation of most NF-κB-regulated genes, and this perfectly fits with the up-regulation of KAT2A being a repressor of NF-κB. Among the down-regulated genes of special interest are the proto-oncogenes Myb and Myc. In normal cells, their expression is tightly controlled for regulating various physiological processes, such as cell proliferation and tissue development. Dysregulation of Myb and Myc has been reported in many cancers [53]. Interestingly, the C-Myb has been found to be down-regulated in NB cells upon induction of differentiation, leading to inhibition of NB cell proliferation [54], and this may suggest its possible role in the onset/development of neuroblastoma tumor. Regarding the Myc family, which comprises three related human genes, a dysregulated expression of n-myc is frequently detected in nervous system tumors such as childhood neuroblastoma, where it represents the strongest predictor of poor prognosis [55]. The observed down-regulation of Myb and Myc in S1.1 cells perfectly aligns with the model of retro-transformation toward a neuronal phenotype being associated with NMD29 over-expression. NDM29 overexpression in human SKNBE2 neuroblastoma cells drives neuronal differentiation, leading to the development of neuritic networks, reduced proliferation, and expression of neuronal genes. It also reduces tumorigenicity *in vivo* and increases sensitivity to chemotherapeutic agents such as cisplatin and doxorubicin, partly through downregulation of MDR1.

Another markedly down-regulated gene is NOS2, the gene coding for the inducible nitric oxide synthase (INOS). Although NOS2 was initially believed to play antitumor activity, recent data revealed that NOS2 expression in cancer cells frequently correlates with a poor prognosis. Indeed, NOS2 expression has been observed to be increased in a wide variety of patient tumors and elevated inducible NOS2 levels have been consistently linked to predicting poor outcomes [56]. In our model, NOS2 was impressively decreased in S1.1 cells, indicating that NDM29 may act also to ameliorate the tumor microenvironment and the inflammatory status. In line with the improvement of the inflammatory status, S1.1 also show a strong reduction in the expression of TNFα and its receptor. TNFα is a pro-inflammatory cytokine triggering and intensifying the body’s inflammatory response to combat infections or other harmful stimuli, but it can also promote tumor growth and progression under certain conditions [57]. Additionally, TNFα can stimulate the production of growth factors that support tumor cell survival and proliferation. Finally, S1.1 cells also presented a decreased expression of the vascular cell adhesion molecule 1 (VCAM1) and the vascular endothelial growth factor (VEGFc). This down-regulation might possibly reduce tumor growth and cancer invasiveness and metastasis [58,59].

Taken together, our data shows a remodeling of genome architecture and epigenome when highly malignant neuroblastoma cells are induced to retro-transform into neuron-like cells by the over-expression of the ncRNA NDM29. This result further strengthens the importance of this ncRNA in NB onset and progression and opens new promising directions for future research in this field. While our findings contribute to a deeper understanding of the molecular mechanisms involved in neuroblastoma malignancy, further studies, particularly those investigating the causa and effect factors sustaining tumor progression, as well as *in vivo* validation, are needed to establish the complete picture of the tumorigenic events. Nonetheless, our results may offer a basis for future development of prognostic markers and therapeutic strategies in NB.

## Supporting information

S1 Fig Resolution of STED. The resolution of the STED images was calculated using the Fourier ring correlation (FRC) assay and is equal to 138 nm.
(PDF)
