# Supplementary figures and images for: Epigenome and three-dimensional genome architecture remodeling during NDM29-mediated retro-transformation of neuroblastoma cells
Source: PLoS One. 2025 Jul 31;20(7):e0327466. doi: 10.1371/journal.pone.0327466 (PMC12312970; doi:10.1371/journal.pone.0327466)

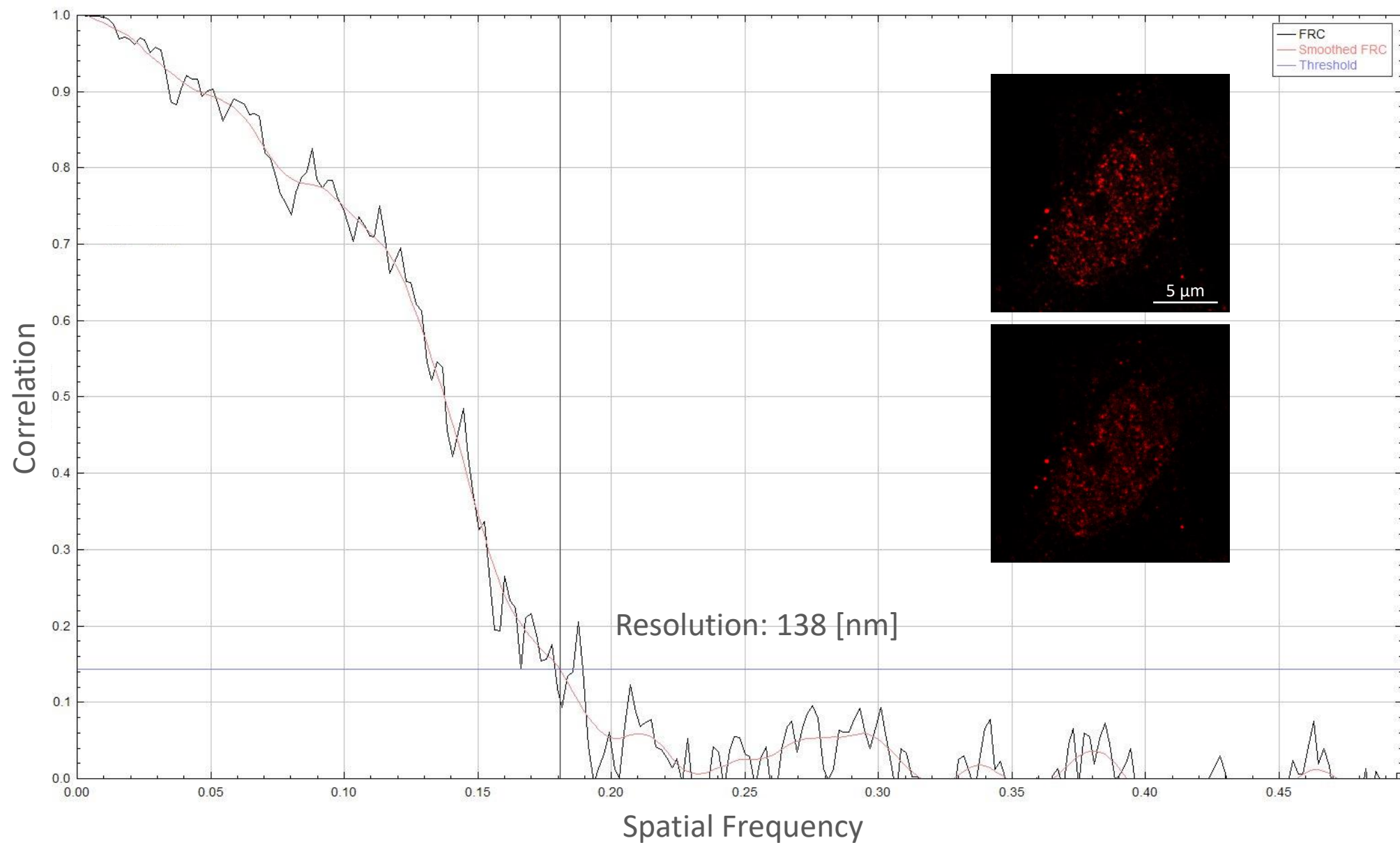

Supplement: S1 Fig — The resolution of the STED images was calculated using the Fourier ring correlation (FRC) assay and is equal to 138 nm. (PDF) [file pone.0327466.s001.pdf]
